# The cost of dual-task walking: Cognitive demands restrict gaze behaviour and gait planning

**DOI:** 10.1371/journal.pone.0337786

**Published:** 2026-04-30

**Authors:** Yuri Russo, Jiaxi Ye, Sarah E. Lamb, Amy Gear, William Lyon, Jinying Qiu, Millie Banks, Emily Martin, Zijing Wang, Maha Alghamdi, Phaedra Leveridge, William R. Young

**Affiliations:** Department of Public Health & Sport Sciences, University of Exeter, Exeter, United Kingdom; University of Muenster, GERMANY

## Abstract

Adaptive walking relies on proactive gaze behaviour to plan foot placement and maintain stability. This study examined how mental workload and task complexity affect gaze behaviour and gait biomechanics during a precision target-stepping task in healthy young adults. We also quantified the frequency of cross-stepping during the experimental task. Twenty-three participants (18–23 years) walked along an L-shaped pathway containing raised stepping targets under single-task (ST) and dual-task (DT) conditions. Targets had four different layouts to create high and low difficulty conditions. Eye movements were recorded using mobile eye-tracking, and gait kinematics were recorded via motion capture. Compared with ST, DT walking produced slower walking speeds, longer stance times, and reduced velocity between stepping targets, indicating a more inefficient gait strategy. In addition, eye-tracking analyses revealed fewer and shorter fixations on task-relevant targets and a greater number of fixations directed toward task-irrelevant areas and, saccadic amplitudes were reduced despite increased outside fixations, suggesting a breakdown in visual exploration between proximal and distal regions of the walkway. In ST conditions, cross-stepping was more frequent than in DT. These findings indicate that increased mental workload compromises proactive gaze behaviour, likely through working-memory and attentional limitations that disrupt feedforward gait planning. Contrary to expectation, cross-stepping occurred more often during ST than DT walking, suggesting that in this population cross-stepping may not be a maladaptive strategy. Overall, these results highlight the cognitive demands of adaptive walking even in young, low-risk individuals and underscore the importance of preserving visual–motor coordination under cognitive stress.

## Introduction

Navigating complex environments safely and efficiently requires us to continuously gather and integrate sensory information. Fixations and saccades are used to maximise the acquisition of visual information by bringing relevant targets from the periphery to the fovea [1]. When walking on even terrains or navigating relatively undemanding environments, advanced visual exploration is typically not necessary for successful locomotion [2]. Thus, we can walk without major problems even when visual function is compromised [3–5]. However, as task complexity and/or environmental demands increase, visual sampling becomes increasingly important for successful task completion [6–10]. Visual Foot Placement Planning (VFPP)—the proactive visual sampling of one’s intended walking route—is essential to adaptive gait as it provides the necessary information to precisely place the feet on areas deemed safe, avoid obstacles, and make ongoing adjustments [11]. Indeed, many studies have reported characteristics of visual fixations and eye movements during adaptive gait as these provide an indication of planning efficiency [7,8,12–14].

When completing complex precision-stepping tasks, young healthy adults typically demonstrate ‘proactive’ VFPP (i.e., visual sampling of stepping constraints located beyond the nearest constraint) [10,15–17]. This proactive strategy allows for effective sampling of the intended walking route so that walkers can make effective motor plans and transition efficiently between stepping constraints. Reductions in proactive VFPP are thought to compromise stepping accuracy and stability [15,18,19]. One potential reason relates to reduced visual information acquired regarding subsequent constraints. Another is the pre-mature re-direction of visual attention away from an intended stepping location prior to the completion of the step [15,20]. In keeping with this, changes in visual search have been interpreted as maladaptive due to the association with increased stepping error.

Walking is not purely automatic, it draws on attentional and executive functions [21], and studies incorporating dual-task (DT) paradigms have shown that when mental workload increases (see [22] for a definition), gait can be disrupted both in young and older adults, although it is most pronounced in the latter [23–25]. DT interference is particularly evident in older adults with worse functional balance and has been linked to increased risk of falling [26–29]. Beyond age-related decline, it is hypothesised that following negative adverse events (e.g., accidents, falls, injuries etc) individuals may start to consciously monitor and control their movements, especially under increased stress or task complexity, redirecting attention towards internal factors and body movements which generally serves to reduce their ability to process external cues [28–30].

Previous research has evaluated the relationship between cognitive demands and VFPP during adaptive gait during a relatively simple walking task [31]. This study showed that DT in young adults did not reduce proactive VFPP but instead induced visual disengagement from the intended walking path. Here, participants showed increased fixations on task-irrelevant areas outside the walkway; a phenomenon the authors referred to as “gazing into thin air”, alongside slower walking speeds and a higher frequency of stepping errors. Similarly, Feld & Plummer reported that participants performing a letter fluency DT (i.e., naming as many words as possible starting with a given letter) directed more gaze toward task-irrelevant regions of a walking path compared to when walking without additional mental workload [32]. Importantly, DT paradigms differ in whether the second cognitive task introduces additional gaze-related demands – i.e., the cognitive tasks differ in the extent to which they necessitate visual fixations or the processing of visuospatial information separately to the walking task. Tasks such as letter fluency, for example, increase cognitive load without specifying where to look and therefore offers a way to evaluate the impact of increased cognitive demands on VFPP and visual disengagement during adaptive gait (i.e., [32]). In contrast, manipulations of motor task complexity (e.g., obstacle negotiation, route selection/turning, and visually guided walking over complex terrain; e.g., [7,8,33]) necessarily impose competing visual sampling demands and therefore constrain VFPP [15]. Together, these findings challenge the assumption that mental workload simply reduces proactive search of task-relevant areas of interest. Instead, they suggest a form of ‘structural interference’, where central executive resources required for the secondary task compete with visual demands for gait planning, resulting in active attempts to visually disengage from the intended walking path to prioritise the processing of a given concurrent cognitive task. While the studies described above have identified the potentially informative phenomenon of task-irrelevant ‘outside’ fixations in the context of gait, their paradigms were not sufficiently complex to evaluate the distribution of visual attention between proximal vs distal stepping constraints. Specifically, Feld & Plummer did not integrate any precision stepping targets and Ellmers and colleagues created stepping targets using tape fixed to the ground with no requirement for specific foot orientation or significant consequences for stepping errors [31,32]. More broadly, current literature describes gaze behaviour during adaptive locomotion, including obstacle negotiation and locomotion over complex/uneven terrain (e.g., [6,8,9,14,16,33]. While these adopted paradigms likely increase attentional demands, they also impose intrinsic visual sampling requirements that can directly constrain or compete for gaze allocation. It therefore remains unclear how added cognitive demands (i.e., a secondary task that does not itself prescribe gaze) influence proactive VFPP when walkers must sample and prioritise multiple, predefined upcoming stepping locations, particularly when accurate foot placement and orientation are required. Despite strong evidence of specific spatiotemporal patterns of proactive visual sampling of planned stepping locations [8,10,17], and that DT disrupts gait [21,25], it remains unclear how concurrent mental workload alters the allocation of gaze across multiple upcoming stepping constraints when accurate foot placement and orientation are required. In particular, previous DT walking studies have typically used relatively simple walking environments or tasks that do not require planning across multiple future targets [31,32], limiting our understanding of how mental workload influences proactive (distal) versus proximal visual sampling during precision stepping. Addressing this gap is essential for understanding how cognitive demands influence VFPP and the visual–motor coordination that supports safe and efficient locomotion in complex environments.

In the current study we aimed to address these gaps by examining how healthy young adults adapt their gaze and gait in response to increased mental workload and a manipulation of task complexity. Using a dual-task paradigm and a target-stepping layout with raised circular targets (i.e., to introducing features that would impact people balance in case of stepping error), we investigated how gaze behaviour strategies and gait biomechanics change under single-task and DT conditions. In this context, we have also measured the frequency of cross-stepping—the medial movement of one foot beyond the position of the other foot relative to the plane of the pelvis—which has been reported as a common cause of falls in older adults and is interpreted here as a risky stepping strategy, even in young healthy adults [34,35]. Therefore, the current study had two main research questions: (1) How does increased mental workload and task difficulty affect proactive gaze behaviour? (2) Do changes in VFPP co-occur with observable costs in gait biomechanics (e.g., reduced speed, increased stance time, increased incidence of cross-stepping) consistent with reduced locomotor efficiency/safety? We anticipate (H1) that DT conditions will provoke a greater number and frequency of outside fixations, even in more complex stepping conditions that increase demand for proactive gaze behaviour of target locations/orientations. We predict (H2) that failure to adequately fixate stepping targets in a proactive manner (i.e., fixating the target more than 2 steps prior to contact) will be associated with increased stepping errors [36,37]. We anticipate that failure to proactively visually sample target locations (i.e., in DT conditions when outside fixations are potentially more prevalent) will be associated with increased incidence of either cross-steps or significant gait inefficiencies (i.e., prolonging stance time, stopping or making additional short steps to avoid cross-stepping) [38].

## Methods

### Study design

This study incorporated a 2x2 within-subjects design (ST vs DT) x (Low vs High difficulty).

### Participants

Twenty-three healthy young adults (age range: 18–23 years old) were recruited and tested between 27/02/2023 and 31/07/2023 from the undergraduate student population at the University of Exeter. Participants were excluded if they had any neurological disorders, visual impairments, or musculoskeletal injuries that could affect gait or balance. Only individuals with corrected-to-normal vision using contact lenses were included; glasses were not permitted. All participants provided written informed consent prior to participation. The study was approved by the internal review board of the Department of Public Health and Sport Sciences, University of Exeter (528055).

### Procedure

**Fig 1:**
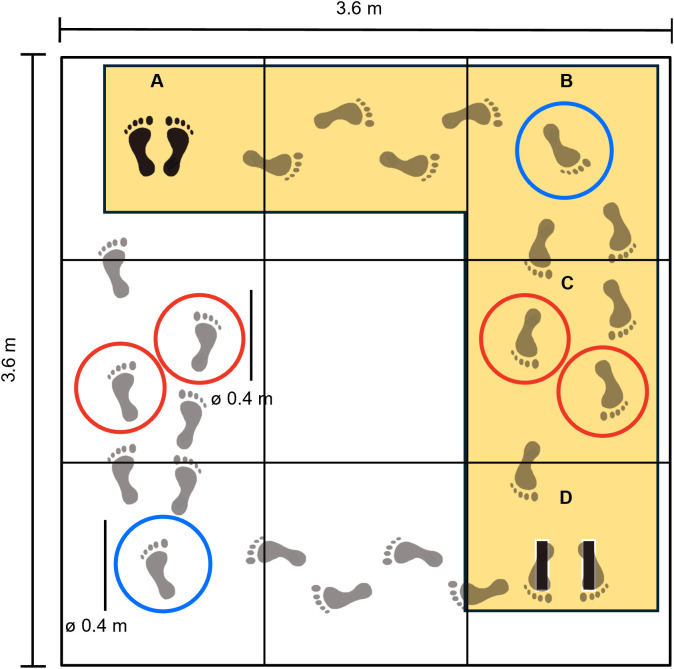
Layout of the experimental protocol. **A** marks the starting point of the walk. Participants began by facing away from the targets B and C to prevent them previewing of the walking path before the start. They then turned and walked straight from point A to point B. At **B**, the blue circle indicates where participants were required to step before turning towards the second straight walk. During this second walk, participants were instructed to step into the red circles (**C**) before stopping with their feet on the marks at **D**, with toes pointing towards the bottom edge of the platform. The exact arrangement of the circles at C varied from trial to trial (see Fig 2). The dark yellow shaded area highlights the L-shaped walkway that defined each trial.

Participants completed a walking task along a predefined path consisting of two straight walkways connected at a 90-degree angle, forming an L-shaped path. Each straight walk measured ~2.5 m (see Fig 1). The start and end points of the task were marked with tape placed approximately at hip width to standardise the initial stance. Participants were instructed to stand on the starting mark facing forward, preventing them from visually pre-scanning the walkway. As they walked, participants were required to step into circular gait orientation targets (raised ~5 cm above ground level) positioned in predefined locations on the walkway. A blue target was located at the turning point between the two straight walkways, and two red targets were placed sequentially on the second straight walkway. Participants completed the walking task barefoot to minimize variability due to footwear [39–41] and were instructed to walk at a comfortable pace, stepping into each target accurately with whichever foot felt natural.

**Fig 2:**
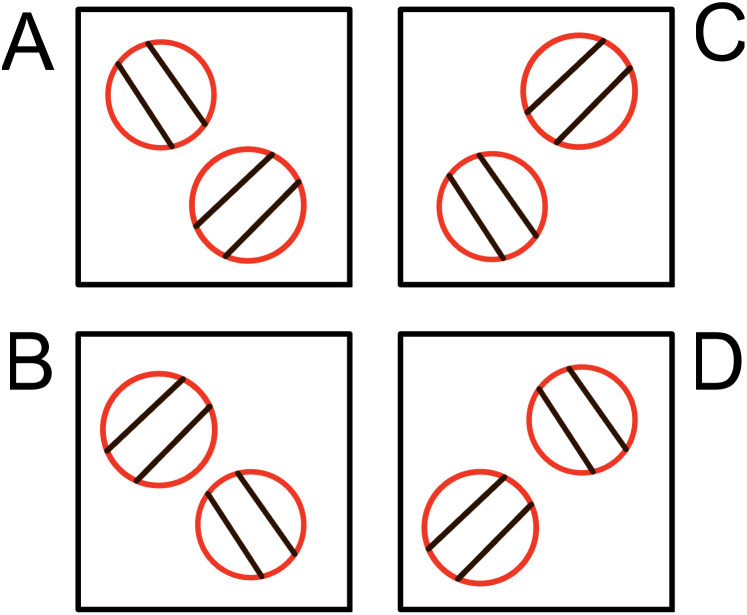
The four possible layouts of the red targets. Layouts **A** and **C** were considered simple because of their outward-oriented configurations. Layouts **B** and **D** were considered difficult due to the inward orientation and positioning of the feet within the targets.

Four different target configurations were used, each representing a different difficulty level (Fig 2). Configurations A and C were considered as relatively simple, characterised by an everted initial stepping target (oriented outward), while configurations B and D were considered difficult, due to an inverted initial stepping target (oriented inward). Foot orientation was visually enforced using plastic strips affixed to the surface of each circular target to indicate the prescribed orientation. These strips provided a clear directional cue for foot placement without using footprint outlines, thereby constraining orientation while minimising explicit limb-specific cues. Target configurations were adjusted at the beginning of each trial while participants stood on the starting mark facing forward, preventing advance visual preview of the walking path.

By varying the position of the initial target, we were able to manipulate whether the natural footfall or walkers be conducive to stepping in the initial target on the ipsilateral side (i.e., right foot in right-sided target) or conducive for cross-stepping (i.e., the natural step length would lead the right foot to the left-side target, or vice-versa). To avoid either cross-stepping or gross stepping inefficiencies (i.e., stopping, making additional steps or rapid adjustments to step length), walkers needed to integrate these prospective requirements into their steps earlier in the approach to the targets shown in Fig 2.

Participants completed the walking task under two conditions: i) single-task (ST), when they had to complete only the walking task; ii) dual-task (DT): when they had to complete the walking task while simultaneously performing a cognitive task involving serial subtraction. Specifically, in the DT condition, participants were asked to subtract 7 multiple times (e.g., 203, 196, 189 etc.) starting from a number given by the experimenter, randomly selected between 185 and 210. We used this cognitive task, to increase the load on working memory and processing load to increase the overall mental workload involved in the walking task. Participants performed the subtraction aloud while walking.

Each participant performed both task conditions in separate, counterbalanced blocks: half of the participants began with the ST condition, and the other half with the DT. Within each block, participants performed multiple repetitions of the walking trial using different target configurations (i.e., simple and difficult). To reduce order effects, the layout and orientation of the gait targets were randomised and changed after every two laps. Each participant completed at least 3 trials in each target layout (Simple vs Difficult) and each condition (ST vs DT).

Before starting the experimental trials, participants were allowed to familiarise themselves with the walking task and DT condition, to ensure they understood the procedure and could perform the task adequately.

### Instruments

Kinematic data were collected using a 19-camera motion capture system (PrimeX 13, OptiTrack, USA) recording at 100 Hz. Each participant wore 12 retro-reflective markers placed on three anatomical landmarks of each foot (heel, lateral malleolus, and head of the first metatarsal), the lateral femoral condyle of each knee, and both the anterior and posterior superior iliac spines. Eye movements were recorded at 100 Hz using an eye-tracking system (Tobii Pro Glasses 2, Sweden), which also captured first-person video of the trials at 25 Hz (1920 × 1080 pixels). Additionally, a video camera (HDR-CX405, Sony, Japan) recorded the trials at 25 Hz to assist with the identification of cross-steps and other gait events.

### Data analysis and outcome variables

Video recordings were reviewed by experimenters (ZW and JQ), who annotated each trial to determine which foot landed in which target and whether a gross stepping error or cross-step occurred.

Kinematic data were labelled, visually inspected, and analysed semi-automatically using custom scripts developed in MATLAB (R2023a, MathWorks, Natick, MA, USA). All kinematic data were low-pass filtered with a fourth-order Butterworth filter using a 15 Hz cut-off frequency (Rum et al., 2023). Individual trials were identified based on spatial thresholds within the motion capture coordinate system. Specifically, a trial was defined as beginning with the participant’s first step motion and ending when the pelvis markers crossed a threshold placed approximately 1 metre in beyond the second red target (see Fig 1).

For each trial, steps were identified based on heel strikes and toe-offs. A heel strike was defined as the minimum vertical position of the heel marker following a local maximum, while toe-off was identified as the minimum vertical position of the toe marker preceding a local maximum.

As target locations remained consistent across trials, their positions in each configuration (A, B, C, D) were approximated graphically in a MATLAB GUI. An experimenter (YR) manually identified specific steps of interest in each trial: the step before the blue target, the step into the blue target, the step into the first red target, and the step into the second red target. These steps were used to compute the following biomechanical outcomes: i) *stance duration* (time from heel strike to toe-off) for the step before the blue target, the step into the blue target, and the step into the first red target; ii) *average velocity* between the blue target and the first red target, calculated as the time between heel strikes on the blue and first red targets; iii) *step interval* time between the first and second red targets, defined as the time between their respective heel strikes. The annotated video data were used to cross-check the accuracy of the detected steps. Additionally, *task duration* was computed as the time from trial start to end, whereas *average walking speed* was calculated by dividing the walking path length (5 m) by task duration.

Eye-tracking data were manually labelled frame-by-frame in Tobii Pro Lab (v1.217) by the experimenters (JY, AG, WL, JQ, MB, EM, MA). Five areas of interest (AOIs) were defined: area before the blue target, blue target, gap, first red target, and second red target. For the analysis, each trial was segmented into two phases: (i) before stepping into the blue target and (ii) after stepping into the blue target. This distinction allowed us to differentiate between instances in which the red targets and the gap were viewed as part of proactive VFPP (i.e., planning future steps) versus proximal VFPP (i.e., guiding immediate foot placement).

### Statistical analysis

Stepping foot into the target (left/right) and the number of steps preceding each target were not treated as experimental factors and were therefore collapsed across trials, such that all trials were included in the statistical analyses regardless of variation in these variables. Data normality was assessed using the Shapiro–Wilk test. Effect sizes were reported as partial eta squared (η²ₚ). A 2 × 2 repeated measures analysis of variance (RM-ANOVA) was conducted to compare biomechanical and eye-tracking variables across cognitive conditions and difficulty levels. All statistical analyses were performed using SPSS (version 28, IBM Corp., Armonk, NY, USA). The significance level was set at α = 0.05 for all tests.

## Results

### Participants

Of the 23 participants recruited, 6 were excluded from the final analysis. Of these, 2/6 participants did not complete the ST condition due to technical issues, and 4/6 had fewer than two valid trials in the DT condition with the difficult target layout. Additionally, 4 participants were excluded from the biomechanical analysis (but not the eye tracking one) due to issues with motion capture recordings. One further participant (PT22) was excluded from biomechanical analyses involving the second red target due to marker visibility issues in the DT condition.

As a result, the final sample included 17 participants for the eye-tracking analysis and 13 participants for the biomechanical analysis. On average, the 17 participants included in the eye-tracking analysis completed 12 ST walks (±5 trials) and 11 DT walks (±5 trials). Variability in the trial count across participants and conditions primarily reflected recording failures and the exclusion of trials that did not meet predefined experimental criteria (e.g., unclear trial boundaries or premature visual preview of the walking path).

### Main outcomes

#### Biomechanical outcomes.

Cognitive task had a significant effect on several biomechanical variables (see Table 1). Trial duration was significantly longer during DT compared ST walking. Consistently, average walking speed was significantly reduced in DT trials, compared to ST.

**Table 1:** Significant differences between conditions for biomechanical outcomes.

Variable	ST (Mean ± SD)	DT (Mean ± SD)	F(1,12)	p	η²ₚ
Trial duration (s)	6.12 ± 1.17	6.69 ± 1.38	8.36	.014	.41
Walking speed (m/s)	0.92 ± 0.18	0.84 ± 0.18	13.13	.003	.52
Stance time Blue target (ms)	577 ± 141	677 ± 126	7.70	.017	.39
Gait Velocity Blue-Red 1 (m/s)	0.92 ± 0.32	0.78 ± 0.29	13.18	.003	.52
Stance time Red 1 target (ms)	618 ± 130	728 ± 151	8.72	.012	.42
Step time Red 1-Red 2 (ms)	620 ± 108	702 ± 144	7.66*	.018	.41

Mean± SD gait parameters are shown for single-task (ST) and dual-task (DT) walking conditions. Effect sizes are reported as partial eta squared (η²ₚ). *Please note that degrees of freedom for step time Red1-Red2 are (1, 11).

Cognitive task also influenced gait parameters associated with specific targets (Table 1). Stance time on the initial blue target was significantly longer in DT than in ST. Similarly, gait velocity between the blue and the first red target was significantly higher in ST compared to DT. Stance time on the first red target was also significantly longer in DT than ST. Finally, the time taken to step between the first and second red targets was significantly greater in DT than ST (Table 1).

No significant differences were found in biomechanical outcomes for task difficulty nor significant task condition x motor difficulty interactions. Statistical results for non-significant effects are provided in the Supplementary Materials (Table S1).

#### Eye-tracking outcomes.

**Fig 3:**
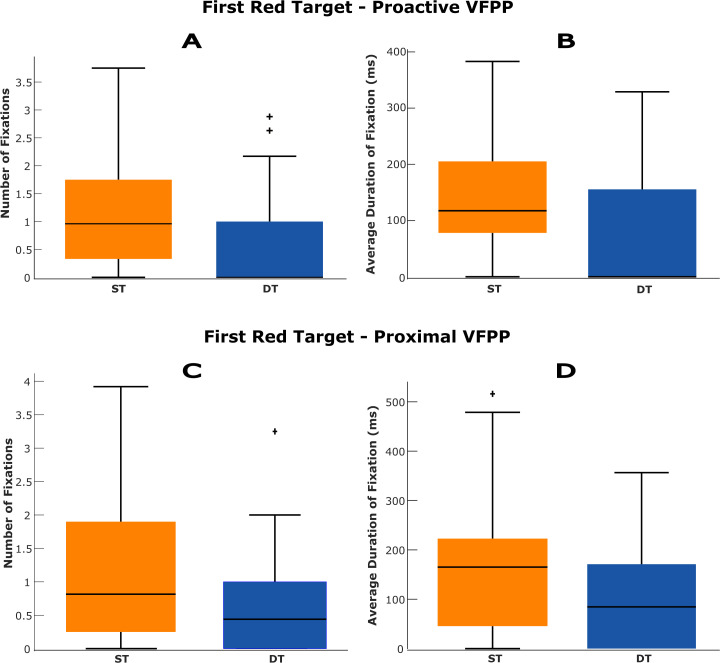
Summary of fixation behaviour (i.e., number of fixations and average fixation duration) on the first red target. Panels A and B show fixation characteristics when the first red target was viewed while approaching the blue target (i.e., proactive VFPP). Panels C and D show fixation characteristics on the first red target after foot contact on the blue target, that is, when the first red target acted as a proximal obstacle along the pathway. ST = single task; DT = dual task; ms = milliseconds.

When examining gaze behaviour on the first red target in in the period when approaching the initial blue target (i.e., the period when fixations on the red targets would be categorised as proactive), both the number and duration of fixations were significantly reduced in DT (Table 2 and Fig 3, top panels). Specifically, DT trials showed a lower number of fixations compared to ST trials, and shorter average fixation durations.

**Table 2:** Significant differences between conditions for eye-tracking outcomes.

Variable	ST Mean ± SD	DT Mean ± SD	F(1,16)	p	η²ₚ
Fixations on red target (approach phase, number)	1.2 ± 0.9	0.7 ± 0.8	10.57	.005	.40
Fixation duration on red (approach phase, ms)	148 ± 92	90 ± 96	5.88	.028	.28
Fixations on red (proximal target, number)	1.2 ± 1.0	0.7 ± 0.7	13.61	.002	.46
Fixation duration on red (proximal target, ms)	174 ± 132	112 ± 87	10.76	.005	.40
Number of Fixations on outside area (number)	3.43 ± 2.1	6.25 ± 3.89	15.29	.001	.49
Saccade amplitude (°)	10.14 ± 1.76	9.07 ± 2.02	8.14	.012	.34

Mean (±SD) gait parameters are shown for single-task (ST) and dual-task (DT) walking conditions. Effect sizes are reported as partial eta squared (η²ₚ).

A similar pattern was observed when the first red target was viewed in the period between foot contact in the first blue target and foot contact in the initial red target (Table 2 and Fig 3, bottom panels). The number of fixations was significantly smaller in DT than in ST. The average duration of fixation on this target was also shorter in DT than ST.

Gaze behaviour on the outside area also differed between cognitive tasks. Participants made significantly more fixations on outside areas during DT trials compared to ST (Table 2). However, there were no significant differences in average fixation duration on the outside area between motor difficulty levels.

Eye-tracking analyses revealed that cognitive tasks significantly affected the average amplitude of saccades, participants made larger saccades in ST than in DT trials.

Notably, a significant condition x motor difficulty interaction emerged for average fixation duration on the outside area, *F*(1,16) = 4.609, *p* = 0.047, η²ₚ = 0.224. In ST trials, fixation duration on the outside area increased from the easy (305 ms ± 44) to the hard layout (404 ms ± 74), while in DT trials, fixation durations remained relatively unchanged (easy: 324 ms ± 42; hard: 303 ms ± 45). No other significant effects of condition or difficulty were observed. Statistical results for non-significant effects are provided in the Supplementary Materials (Table S2).

### Secondary outcomes

#### Descriptive analysis of cross-steps.

A total of 57 cross-steps were observed across the 17 participants included in the analysis. Of these, 13 participants exhibited cross-stepping behaviour when stepping onto the red targets, with a range of 1–11 cross-steps per participant (median = 3; interquartile range = 8.5). Descriptively, a greater frequency of cross-step events was observed during single-task trials (n = 43) than during dual-task trials (n = 14). Due to the heterogeneous distribution of cross-steps across participants and across conditions, inferential statistical comparisons between cross-steps and standard steps were not performed. Descriptive biomechanical characteristics of cross-steps and non-cross steps in ST and DT conditions are presented in Table 3.

**Table 3:** Overview of the biomechanical outcome measures, divided according to task type (single vs. dual task) and the presence of cross-stepping.

	Trial Duration (s)	Average Velocity (m/s)	Stance Before Blue (ms)	Stance in Blue (ms)	Velocity between blue and red (m/s)	Stance in 1^st^ Red (ms)	Stance in 2^nd^ Red (ms)
ST Cross-Step	5.88 ± 1.25	.93 ± .20	639 ± 127	563 ± 154	.92 ± .29	617 ± 205	640 ± 170
ST Non-Cross-Step	6.59 ± 2.24	.81 ± .17	664 ± 133	643 ± 145	.92 ± .30	666 ± 224	644 ± 153
DT Cross-Step	6.07 ± 1.24	.86 ± .17	699 ± 174	694 ± 238	.73 ± .20	679 ± 174	651 ± 257
DT Non-Cross-Step	6.87 ± 1.63	.77 ± .16	690 ± 145	693 ± 163	.79 ± .33	748 ± 154	725 ± 195

ST = single task; DT = dual task.

## Discussion

This study investigated how mental workload and task difficulty influence gaze behaviour and gait biomechanics in healthy young adults during a target-stepping task. Our results show that cognitive dual-tasking served to significantly restrict gaze behaviour and influenced locomotor performance, providing new insights into the effects of cognitive demands on adaptive gait in young populations.

### Dual-tasking reduces proactive gaze behaviour and motor performance

Participants walked more slowly and exhibited longer stance times when engaged in a DT compared to a ST, consistently with our initial hypothesis and the broader literature showing that mental workload impairs motor performance [21,24,31]. Reduced velocity between stepping targets and prolonged stance times may indicate that participants adopted a more cautious gait strategy under additional mental workload, possibly a compensation for disruptions in feedforward planning and therefore reduced visual-motor coordination. One alternative explanation is that slower walking provides more time to sample visual information from the surroundings. However, eye-tracking data showed concurrent changes in gaze allocation that further support the first hypothesis. Under DT conditions, participants fixated less frequently and for shorter durations on task-relevant areas, especially the first red target, and directed their gaze more towards task-irrelevant outside areas. This shift in gaze behaviour is consistent with reduced proactive VFPP, which is critical for feedforward motor planning. These findings corroborate previous observations that additional mental workload induced “gazing into thin air”, a form of active visual disengagement from the walking task [31]. While prior work has shown that increasing cognitive demands can alter gaze allocation during walking (e.g., increased task-irrelevant fixations and reduced sampling of farther regions of the environment [[40],[32]]), the present study extends this literature by providing direct evidence that DT restricts proactive sampling of distal stepping targets in a multi-target precision stepping task. When considering these findings in conjunction with our own subjective evaluation of the data, we suggest that increased cognitive demands appear to influence visual and stepping behaviour in three main ways. First, walkers reduced the extent to which they fixate future stepping constraints on the intended route, especially those beyond the immediate constraint. Second, DT-related visual disengagement from the intended path was typically characterised by fixations that appear to be excessively displaced from task-relevant features. Here, smaller saccades and associated fixations often occur around a single task-irrelevant outside area while the walker processed the next iteration of the arithmetic task (consistent with [31]). Subsequent large amplitude saccades were not typically made unless returning fixations to the walkway. Third, fixations were generally shorter in the DT condition. For those fixations on the targets, shorter fixation durations appeared to be driven by the strategy to visually disengage from the walking route once sufficient visual information regarding the targets had been sampled.

DT reduced mean saccade amplitude, alongside a clear increase in the number of outside fixations and a concurrent reduction in both the number and duration of fixations on the stepping targets during both proactive and proximal phases (see Table 2). This indicates restricted VFPP at points where walkers would ordinarily acquire and update visuospatial information to plan upcoming steps. One plausible explanation, consistent with prior descriptions of “gazing into thin air” [31], is that participants disengaged from the walkway to a relatively stable, task-irrelevant location and then produced smaller saccades around a specific area outside the walking route, which could serve to simultaneously reduce mean saccade amplitude while increasing the frequency of outside fixations [1,2].

The reduction in the number and duration of stepping-target fixations during both proactive and proximal phases (see Table 2) suggests that increased cognitive demands can jeopardise the ability to acquire, and potentially retain, task-relevant visuospatial information about multiple upcoming stepping constraints [42]. This offers insights to previous work in anxious or at-risk walkers, where reduced VFPP has often been interpreted in relation to conscious movement processing (CMP) and increased reliance on online control [18]. In applied contexts, the current results show that task-irrelevant cognitive demands alone (i.e., added mental workload without introducing additional visual targets) are sufficient to restrict proactive target sampling during multi-target precision stepping. This also highlights the potential relevance of internal, task-irrelevant processes (e.g., worry-related thoughts) as factors that may reduce proactive planning in everyday walking contexts [36].

In parallel, DT reduced mean saccade amplitude alongside an increase in the number of outside fixations (Table 2). One plausible interpretation, consistent with prior descriptions of “gazing into thin air” [31] and the VFPP changes described above, is the intermittent disengagement of gaze to a relatively stable off-walkway location and/or fewer transitions between task-relevant regions, both of which could reduce mean saccade amplitude while increasing outside-fixation frequency. However, because we analysed mean saccade amplitude only (rather than saccade-size distributions or AOI-to-AOI transitions), we cannot distinguish between these alternative scan path mechanisms. Conceptually, this pattern aligns most closely with distraction-based accounts and associated competition for resources (e.g., attentional control frameworks in which task-irrelevant processing reduces goal-directed allocation of attention [43]), while remaining compatible with self-focus accounts used to interpret CMP-related changes in adaptive gait; importantly, these mechanisms are not mutually exclusive and require targeted testing in future work.

Finally, the reduction in fixations on the first red target following blue-target contact under DT further supports the interpretation that increased cognitive demands constrained proximal visual sampling at a point when accurate stepping adjustments would ordinarily be supported by targeted visual updates. Because the secondary task did not impose additional gaze demands, this reduction is unlikely to reflect direct visual competition from the secondary task and instead implicates limitations in cognitive resources supporting search, retention, and updating of task-relevant visuospatial information.

Previous work has also shown that restrictions in VFPP are associated not only with increased stepping errors but also with longer stance phase durations around precision steps [36]. The present findings further support this suggested link. A failure to proactively acquire visuospatial information about upcoming target locations and orientations is likely to compromise feedforward movement planning during the approach to the targets. As a result, walkers would slow down, particularly during the critical phases of gait where planning of future steps and propulsion of the centre of mass are carried out. However, our findings – and those of previous studies [31] – suggest that participants may still attempt to maintain overall walking velocity despite these visuomotor constraints. In such cases, walkers appear to compensate by prolonging critical stance phases (e.g., stance in the blue target) rather than reducing speed substantially, a strategy that may preserve forward momentum but at the cost of reduced stepping accuracy (see also studies on anxiety and cognitive-motor interference [37,44]).

### Outside fixations may be indicative of visuomotor ‘Spare Capacity’

In line with our hypothesis, participants produced more outside fixations during the DT condition, compared with ST. Consistent with previous reports of this behaviour [31], this finding appears to represent a structural interference between the motor task of walking and concurrent cognitive task. However, the current study helps to progress our understanding of this phenomenon due to the additional manipulation of motor task complexity. One might expect that increasing demands of the motor task – thereby increasing the importance of proactively scanning the red targets – would restrict visual disengagement from the walkway. Our results showed no significant effect of motor task difficulty on gaze behaviour directed toward the red targets. However, the duration of outside fixations increased in the more complex motor task, but only during ST conditions. This finding directly contradicts our initial predictions and highlights the need to consider the origins and functions of outside fixations. Our results support the notion that high cognitive demands can be sufficient to induce outside fixations by virtue of an active disengagement that is presumably intended to avoid visual distractions associated with movement planning while prioritising the cognitive task. However, the same rationale cannot be applied to the ST condition. Here, the longer outside fixation durations are unlikely to be driven by any increased cognitive demands of processing information relating to the more complex target locations. We speculate that, upon recognising a more complex arrangement of targets, walkers developed an implicit understanding that there will be greater demands for motor planning in the step preceding the red targets. Once the constraints of the upcoming task are understood, the walker is at liberty to visually explore the path or temporarily disengage without cost to the motor task. In this sense, we recommend that outside fixations should be considered as an expression of ‘spare capacity’ in the visuomotor system.

It is important to note that the current results suggest that in more difficult gait tasks, cognitive DT is not sufficient to drive outside fixations. In other words, due to the added requirements for movement planning, there is less capacity to disengage the gaze from the walkway. However, in the ST high difficulty tasks we observed more outside fixations. This contradicts the notion that increased requirements for movement planning are negatively associated with outside fixations and instead implies that those requirements might predispose people to visually disengage. Previous reports of outside fixations interpret it as a strategy to temporarily prioritise the cognitive task by avoiding task-relevant fixations [31]. In the current task, the adaptive gait task was far more challenging compared to previous studies, especially around the red targets in the hard condition. This raises the possibility that, upon recognising the harder arrangement of red targets, walkers engaged in a strategy to retain this information, and this emerged as visual disengagement from task-relevant areas during periods of the task where precision stepping was not required. These mechanisms are likely to also have been present in DT conditions. However, we speculate the greater number of outside fixations observed in DT conditions is likely to have masked this effect. This is likely to have been particularly true in the easy target condition, as outside fixations is already shown to be increased by DT in easier gait tasks [31,35].

### Cross-stepping as an adaptive and context-dependent strategy

Contrary to our initial hypothesis, we observed a higher frequency of cross-steps in the ST condition compared to the DT condition. Cross-stepping is often interpreted as a challenging compensatory strategy, typically associated with spatial or timing constraints and thought to reflect inefficiencies in gait planning [45]. However, in this context, we propose an alternative explanation. In ST trials, participants were likely able to allocate more cognitive resources to the motor task, allowing them to explore a broader range of stepping strategies, including cross-steps, without compromising dynamic balance. Although the raised targets required increased precision for foot placement, they may not have represented a substantial threat to balance in this population. As such, cross-stepping may have been perceived as a safe and efficient solution under ST conditions, as suggested by the observed velocities reached during the different tasks (Table 3). In contrast, under DT conditions, the added mental workload may have reduced participants’ confidence in adopting a biomechanically more challenging strategy. This could have led to a more conservative approach, favouring the adoption of normal stepping strategies that required less attentional regulation and minimised perceived risk. These preliminary findings challenge the notion that cross-stepping is inherently maladaptive and suggest that the selection of stepping strategy could be context and population dependent and influenced by perceived motor capacity. Because cross-stepping was analysed descriptively in the present study, future research should evaluate its functional role and perceived risk using designs that can support inferential comparisons across different populations and walking environments. This is particularly important in older adults and individuals with impaired balance, and under varying cognitive and physical demands.

### Limitations

Several limitations should be acknowledged. First, the sample was limited to healthy young adults, and findings may not be generalised to older or clinical populations who exhibit different gait and cognitive abilities. Second, although the study used a well-controlled target-stepping paradigm, real-world walking environments are more variable and unpredictable. Third, due to the uneven and limited distribution of cross-step events, we were unable to perform inferential comparisons between cross- and standard stepping behaviours. Finally, as our saccade analysis used mean amplitude, the current data cannot distinguish between different scan path mechanisms (e.g., occasional large disengagement saccades followed by many small saccades vs. fewer large target-updating saccades overall). Future work should therefore quantify saccade amplitude distributions (e.g., proportion of large saccades) and AOI-to-AOI transition patterns to characterise how cognitive load reshapes the temporal sequencing of gaze updates during precision stepping.

## Conclusions

This study demonstrates that dual-tasking compromises both gaze behaviour and gait performance in healthy young adults, particularly by reducing proactive gaze behaviour and slowing stepping dynamics. These results highlight the cognitive demands of adaptive walking, even in low-risk populations, and underscore the importance of visual attention in locomotor planning. By identifying how mental workload disrupts the spatial and temporal allocation of gaze during walking, this work contributes to a deeper understanding of attentional control during movement and informs the development of interventions aimed at enhancing safety in more complex and cognitively challenging environments.

### Highlights

- Mental workload during walking restricts proactive gaze behaviour and alters gait dynamics in healthy young adults.
- Dual-tasking led to slower walking speeds, longer stance times, and reduced step-to-step velocity.
- Eye-tracking showed fewer and shorter fixations on stepping targets, and more gaze directed toward task-irrelevant areas under dual tasking.
- Reduced proactive gaze under dual-task conditions likely reflects limitations of available cognitive resources rather than task-specific prioritisation.
- Findings highlight how cognitive demands compromise visual–motor coordination, supporting interventions that promote gait automaticity and reduce mental workload.
- Cross-stepping was more frequent in ST than in DT conditions, suggesting that cross-stepping may not be maladaptive in healthy young people.

## Supporting information

S1 Table Comparisons between target difficulty conditions for biomechanical outcomes. Values are mean ± SD for simple and difficult target configurations. Reported statistics correspond to the main effect of difficulty from the 2 × 2 RM-ANOVA; all effects shown in this table were non-significant (p ≥ 0.05). Effect sizes are reported as partial eta squared (η²ₚ). *Degrees of freedom for step time (Red1–Red2) are (1, 11).
(DOCX)

S2 Table Comparisons between target difficulty conditions for eye-tracking outcomes. Values are mean ± SD for simple and difficult target configurations. Reported statistics correspond to the main effect of difficulty from the 2 × 2 RM-ANOVA; all effects shown in this table were non-significant (p ≥ 0.05). Effect sizes are reported as partial eta squared (η²ₚ).
(DOCX)
