# Supplementary material for: The cost of dual-task walking: Cognitive demands restrict gaze behaviour and gait planning
Source: PLoS One. 2026 Apr 30;21(4):e0337786. doi: 10.1371/journal.pone.0337786 (PMC13132448; doi:10.1371/journal.pone.0337786)
Supplement: S1 Table — Values are mean ± SD for simple and difficult target configurations. Reported statistics correspond to the main effect of difficulty from the 2 × 2 RM-ANOVA; all effects shown in this table were non-significant (p ≥ 0.05). Effect sizes are reported as partial eta squared (η²ₚ). *Degrees of freedom for step time (Red1–Red2) are (1, 11). (DOCX) [file pone.0337786.s001.docx]

| **Variable** | **Simple**  **(Mean ± SD)** | **Difficult**  **(Mean ± SD)** | **F(1,12)** | **p** | **η²ₚ** |
| --- | --- | --- | --- | --- | --- |
| Trial duration (s) | 6.4 ± 1.3 | 6.5 ± 1.3 | .003 | .955 | >.001 |
| Walking speed (m/s) | .88 ± .19 | .88 ± .18 | .204 | .660 | .017 |
| Stance time  Blue target (ms) | 620 ± 133 | 636 ± 147 | 1.199 | .295 | .091 |
| Gait Velocity  Blue-Red 1 (m/s) | .81 ± .27 | .90 ± .37 | 4.683 | .051 | .281 |
| Stance time  Red 1 target (ms) | 674 ± 157 | 672 ± 144 | .006 | .942 | >.001 |
| Step time  Red 1-Red 2 (ms) | 659 ± 131 | 664 ± 125 | .104 | .753 | .009 |

Table S1
