# Supplementary material for: The cost of dual-task walking: Cognitive demands restrict gaze behaviour and gait planning
Source: PLoS One. 2026 Apr 30;21(4):e0337786. doi: 10.1371/journal.pone.0337786 (PMC13132448; doi:10.1371/journal.pone.0337786)
Supplement: S2 Table — Values are mean ± SD for simple and difficult target configurations. Reported statistics correspond to the main effect of difficulty from the 2 × 2 RM-ANOVA; all effects shown in this table were non-significant (p ≥ 0.05). Effect sizes are reported as partial eta squared (η²ₚ). (DOCX) [file pone.0337786.s002.docx]

| **Variable** | **Simple**  **Mean ± SD** | **Difficult**  **Mean ± SD** | **F(1,16)** | **p** | **η²ₚ** |
| --- | --- | --- | --- | --- | --- |
| Fixations on red target  (approach phase, number) | .95 ± .98 | .93 ± .86 | .036 | .852 | .002 |
| Fixation duration on red  (approach phase, ms) | 114 ± 103 | 125 ± 100 | .519 | .482 | .033 |
| Fixations on 2^nd^ red target  (approach phase, number) | .32 ± .52 | .30 ± .34 | .081 | .779 | .005 |
| Fixations on red  (proximal target, number) | .89 ± .97 | .95 ± .93 | .325 | .577 | .020 |
| Fixation duration on red  (proximal target, ms) | 169 ± 85 | 199 ± 130 | 1.688 | .218 | .123 |
| Fixations on 2^nd^ red  (proximal target, number) | 1.22 ± .90 | 1.35 ± 1.0 | 1.832 | .195 | .103 |
| Fixation duration on 2^nd^ red  (proximal target, ms) | 162 ± 76 | 191 ± 106 | 2.040 | .177 | .136 |
| Number of Fixations  on outside area (number) | 2.38 ± 1.17 | 2.26 ± 1.17 | .878 | .363 | .052 |
| Saccade amplitude (°) | 10.2 ± 3.1 | 9.2 ± 3.3 | 2.953 | .105 | .156 |
